# TRIB2 contributes to cisplatin resistance in small cell lung cancer

**DOI:** 10.18632/oncotarget.22741

**Published:** 2017-11-27

**Authors:** Yuanxin Liang, Dong Yu, Roman Perez-Soler, Jim Klostergaard, Yiyu Zou

**Affiliations:** ^1^ Department of Medicine/Cancer Center, Albert Einstein College of Medicine, Bronx, NY, USA; ^2^ Department of Respiratory and Oncology, Hubei Provincial Corps Hospital, Wuhan, China; ^3^ Department of Molecular and Cellular Oncology, MD Anderson Cancer Center, Houston, TX, USA; ^4^ Department of Pathology, Tufts Medical Center, Boston, MA, USA

**Keywords:** TRIB2, CEBPA, small cell lung cancer, cisplatin, resistance

## Abstract

Small cell lung cancer (SCLC) is the most aggressive lung-cancer subtype and so far, no favorable therapeutic strategy has been established for chemo-resistant SCLC. Cisplatin is one of the most important components among all standard poly-chemotherapeutic regimens for SCLC; therefore, this study focused on revealing Cisplatin-resistance mechanism(s) in this disease. Cisplatin-resistant SCLC cells were generated in the NCI-H69 xenograft model in nude mice by continuous intravenous administration of Cisplatin; Cisplatin resistance of the tumor cells was confirmed by *in vitro* and *in vivo* tests, and the gene expression profile of the resistant cells was determined using microarray analysis. A significantly higher expression of tribbles pseudokinase 2 (TRIB2) mRNA in the Cisplatin-resistant cells was found compared to parental H69 cells. Further, the Cisplatin-resistance level was decreased when TRIB2 expression was knocked down. The mRNA and protein levels of CCAAT/enhancer binding protein alpha (CEBPA), known to be a transcription factor regulating cell differentiation and a target for degradation by TRIB2, as well as selected cancer stem cell makers in the Cisplatin-resistant cells, were measured. We found that CEBPA protein levels could be upregulated by knocking down the overexpressed TRIB2, which also reversed the Cisplatin-resistance of these cells; further, the Cisplatin-resistant SCLC cells demonstrated certain cancer stem cell-like properties. Similar patterns were also observed in limited human tumor specimens of chemo-resistant SCLC patients: namely, overexpressed TRIB2 and undetected CEBPA proteins. Our study revealed a possible molecular mechanism for Cisplatin-resistant SCLC involving induced TRIB2 overexpression and downregulation of CEBPA protein. We propose that this mechanism is a potential therapeutic target to circumvent chemo-resistance in SCLC.

## INTRODUCTION

Small cell lung cancer (SCLC) is the most aggressive lung-cancer subtype with only rare long-term survival [1] [2]. Despite generally showing an excellent response to initial chemotherapy, most SCLC patients experience relapse within 2 years, with a refractory relapse that is chemo-resistant and with abysmal prognosis [3] [4]. Unfortunately, no favorable therapeutic strategy has been established in recurrent, chemo-resistant SCLC [5]; thus, it is very important to define these chemo-resistance mechanisms and thereby identify new therapeutic targets.

Several abnormalities have previously been found contributing to these chemo-resistance mechanisms. These include 1) dysregulation of apoptosis linked to Bcl-2, 2) extracellular matrix (ECM)-mediated anti-apoptotic effects on SCLC cells, and 3) acquisition of cancer stem cell (CSC)-like properties.

Overexpression of Bcl-2 was found to be parallel to Cisplatin resistance, and bcl-2 stable transfection strongly reduced Cisplatin sensitivity in SCLC cells [6]. Osteopontin stable transfection resulted in a higher ratio of Bcl-2/Bax and lower activation of caspase-9 and caspase-3 following Cisplatin treatment [7]. Bcl2-associated athanogene 3 (BAG3) expression was detected in SCLC cell lines as well as in SCLC patient samples, and siRNA down-regulation of BAG3 sensitized SCLC cells to Cisplatin treatment [8].

ECM-mediated protection of SCLC cells from chemotherapy-induced apoptosis has been reported. For instance, adhesion to ECM stimulated protein tyrosine kinase (PTK) activation through beta1 integrins, and the activated PTK blocked the activation of caspase-3 and subsequent apoptosis [9].

It was reported that hepatocyte growth factor (HGF) mediated epithelial-to-mesenchymal transition, and the induced expression of mesenchymal markers was associated with chemo-resistance, which could be reversed by Met (a HGF receptor) inhibitor. Further, CSC markers were associated with Met activation in human SCLC specimens, and they were predictive for worse survival and associated chemo-resistance [10].

Although the previous studies contributed to our understanding of the basis for chemo-resistance by manipulating the expression of specific genes, these were done without naturally reproducing the Cisplatin-resistance phenotype; therefore, some of the key factors representing the practical development of chemo-resistance in SCLC patients may have been missed, and thus the pathways mediating chemo-resistance remain largely unknown [11].

Cisplatin is one of the most important components in standard poly-chemotherapy regimens for SCLC [12], [13]; therefore, we investigated gene expression changes associated with Cisplatin-resistance as one approach to reveal relevant chemo-resistant mechanism(s). We implanted NCI-H69 SCLC cells subcutaneously [14] into nude mice and treated the mice with multiple intravenous injections of Cisplatin for one year. Next, we isolated SCLC cells from these tumors, and compared them with the original, parental H69 cells. We verified that the Cisplatin-treated tumor cells were indeed much more resistant to Cisplatin *in vitro* and determined that the TRIB2 gene expression levels increased significantly. TRIB2 has previously been identified as an oncogene that causes acute myelogenous leukemia via inactivation of the transcription factor, CEBPA (CCAAT/enhancer-binding protein alpha) [15], [16]; however, to date there is no established relationship of TRIB2 with chemo-resistance. In this study, we investigated one underlying molecular mechanism between unregulated TRIB2 and Cisplatin-resistance in both the Cisplatin-resistant SCLC cell model and in pilot studies of human SCLC tumor specimens.

## RESULTS

### Cisplatin resistance was confirmed *in vitro*

To date, although a Cisplatin-treated SCLC xenograft model has recently been reported [17], there has been no single ideal animal model accurately simulating development of Cisplatin resistance *in vivo*, which has greatly handicapped studies on mechanisms of such resistance. Thus, to mimic this process, we implanted multiple SCLC cell lines (H82, H446, H660, H345 and H69) into nude mice to develop a Cisplatin-resistant model. Cell line selection criteria are explained in Supplementary Data 7. Among all these models, the H69 xenograft was the only one which tolerated long-term Cisplatin-treatment and developed resistance compared to the tumor established from the parental cells. We collected and cultured the SCLC cells from the H69-implanted mice at 6, 9, and 12 months after Cisplatin-treatment, and found that only the cells from 12-month treated mice demonstrated significantly higher resistance than untreated parental H69 cells.

**Figure 1:**
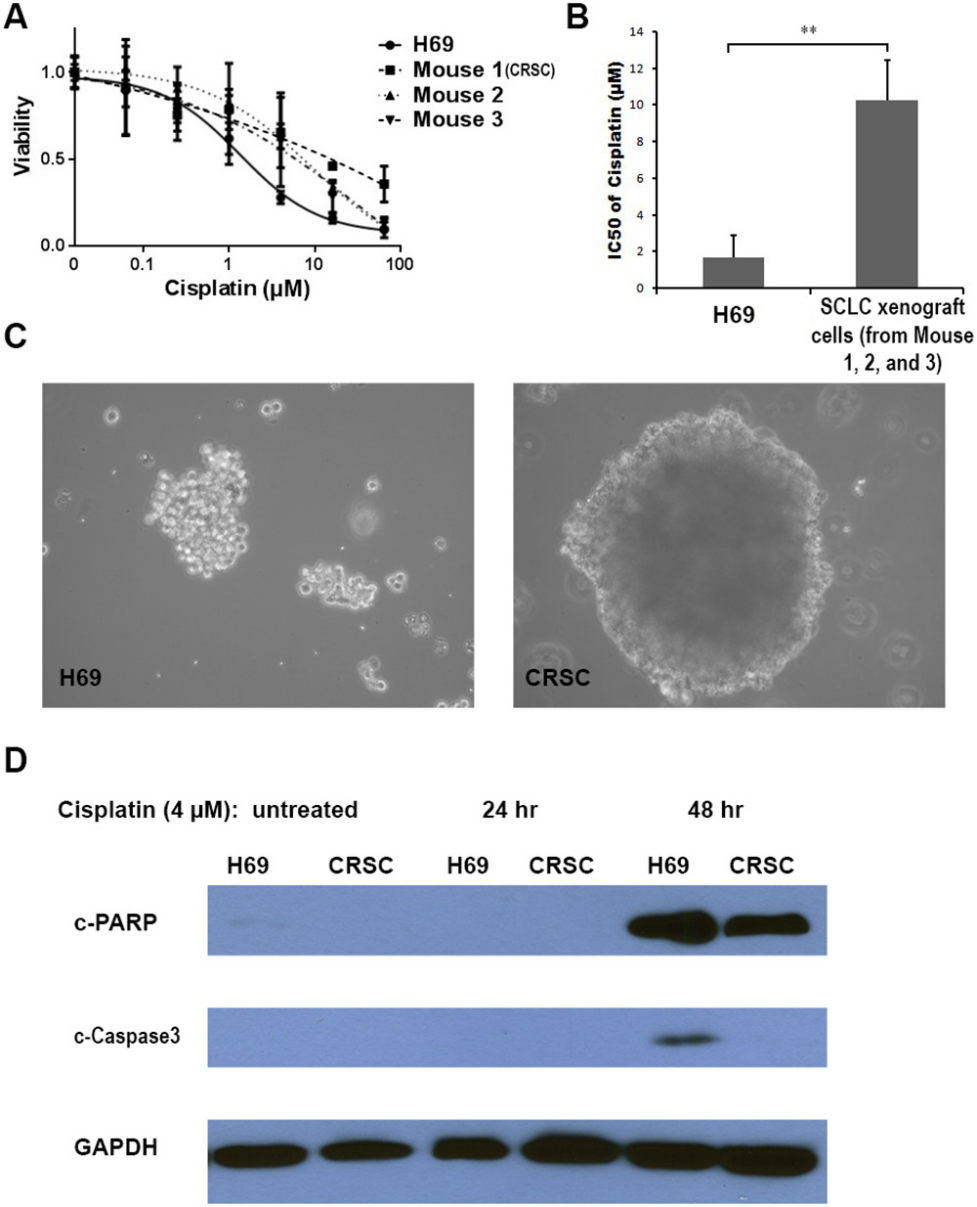
*In vitro* Cisplatin resistance of Cisplatin-selected SCLC cells **(A)** Cytotoxic effects of Cisplatin in the H69 cells and the SCLC cells from Cisplatin-treated mice. **(B)** Average IC_50_s of Cisplatin in H69 cells and in SCLC cells from Cisplatin-treated mice. **(C)** Morphology of H69 and CRSC cells (×200). **(D)** Immunoblot analysis of apoptotic markers during Cisplatin treatment in H69 and CRSC cells. (^**^, P < 0.01).

In order to verify that the s.c.-implanted H69 tumor cells became Cisplatin-resistant, we harvested the SCLC tumors from three mice and cultured them separately. By applying an accurate cytotoxicity assay [18], we found the IC_50_ of Cisplatin in the SCLC cells isolated from the Cisplatin-mice was 7.1-, 6.5-, and 4.6-fold higher than that of parental H69 cells (Figure 1A): the average IC_50_ from the Cisplatin treated mice was 10.26 ± 2.16 μM, while the IC_50_ of H69 cells was 1.69 ± 1.22 μM (p<0.01) (Figure 1B). The cells from mouse 1 showed the highest IC_50_ of Cisplatin (11.98 μM) and were named CRSC (Cisplatin Resistant SCLC Cells) for further study. Morphological differences between the resistant and sensitive cells were also noted; CRSC cells formed larger clusters than parental H69 cells in culture (Figure 1C).

We further evaluated the Cisplatin-resistance by measuring the apoptotic level of CRSC and H69 cells, as the apoptotic mechanism has previously been established. Cisplatin induces cell death via apoptosis through activation of the intrinsic mitochondrial pathway and extrinsic death receptor pathway [19], and cleaved caspase-3 and PARP are considered ideal markers for evaluating this apoptotic process [20] [21]. These apoptotic markers have previously been used to measure the apoptosis levels induced by Cisplatin-treatment in lung cancer cells [22]. After treatment with 4 μM Cisplatin for 48 hrs, cleaved caspase-3 was detected in H69 cells, but not in CRSC cells; further, higher levels of cleaved PARP were evident in treated H69 cells compared to CRSC cells (Figure 1D). These *in vitro* results indicate that SCLC cells from Cisplatin-treated mice were more apoptotically-resistant to Cisplatin than parental H69 cells. (Figure 1).

### Cisplatin-resistance was confirmed *in vivo*

**Figure 2:**
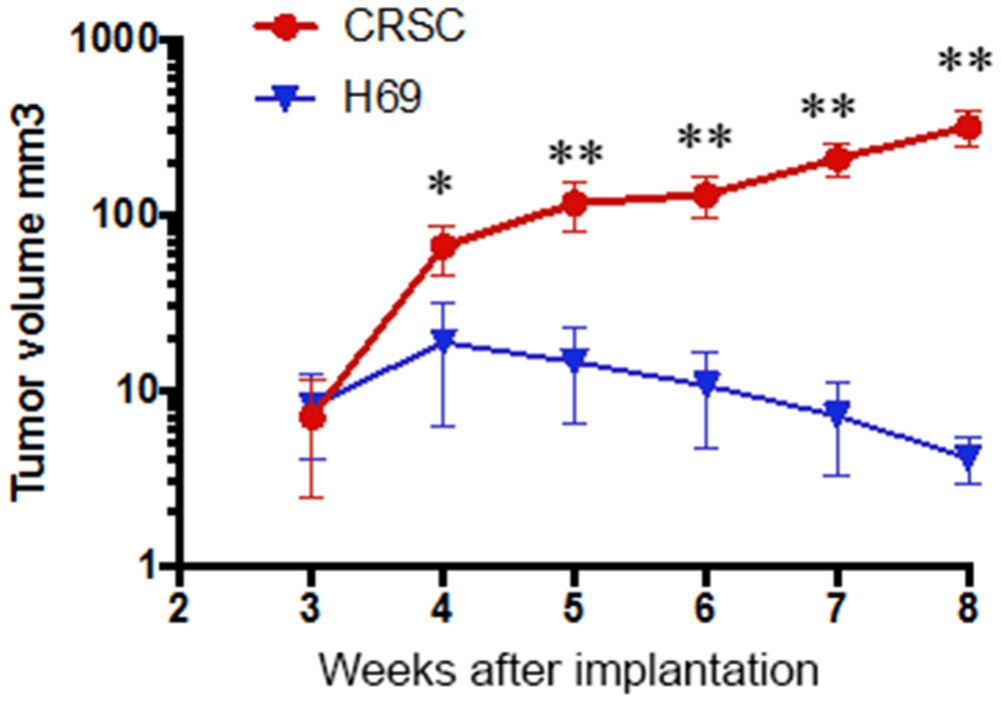
*In vivo* Cisplatin resistance of Cisplatin-treated SCLC cells The plot shows the tumor volume from the SCID mice implanted with H69 and CRSC cells, Cisplatin injections started 3 weeks after the implantation. (^*^, P < 0.05, ^**^, p < 0.01, H69 vs. CRSC).

In order to further confirm the resistance of CRSC *in vivo*, we directly compared the CRSC and H69 tumor responses to Cisplatin in SCID mice [23] in two separate tests. In one test, the H69 and CRSC cells (2x10^6^ cells/mouse) were separately implanted into two groups of mice. When the average size of tumors reached ∼ 2-3 mm in diameter (about 3 weeks), the mice received three weeks of i.v. Cisplatin treatment. The tumor volume ratios of CRSC vs. H69 were 7.0 ± 4.6 vs. 8.1 ± 4.1 mm^3^ (p = 0.6855) when the treatment started (week 3), 130.5 ± 33.9 vs. 10.6 ± 6.0 mm^3^ (p < 0.0001) when the treatment ended (week 5), and 426.9 ± 105.3 vs. 1.9 ± 1.1 mm^3^ (p < 0.0001) when the test ended (week 8). The average body weights of the two groups at the same time points were indistinguishable: 24.4 vs. 24.6, 19.8 vs. 19.9, and 20.4 vs. 20.6 g (Figure 2). In another test, equal numbers of H69 and CRSC cells were simultaneously inoculated s.c. into the right and left flanks, respectively, of the same mice to ensure that the cells grew in similar environments and received the same treatment dose. CRSC cells grew out in 5/5 (100%) mice and H69 cells did in 4/5 (80%) mice. After a three-week treatment protocol with seven Cisplatin injections, the average body weight of the mice decreased from 36.5 to 29.4 g (p < 0.01), the average volume of H69 tumors became undetectable, dropping from 5.9 to 0 mm^3^ (p < 0.01) (no tumors still detectable); however, CRSC tumor diameters increased from ∼4.8 mm to ∼12.3 mm in spite of the high-dose Cisplatin treatment. Obvious tumors of CRSC cells were observed on the left flanks of the mice upon sacrifice, whereas no H69 tumors were found on the right flanks of the same mice (Supplementary Data 6). Collectively, our data from isolated tumor cells *in vitro* (Figure 1) and from the tumors in mice (Figure 2) consistently demonstrated that the CRSC were significantly Cisplatin-resistant. These *in vivo* Cisplatin-induced resistant tumors partially mimicked the process of platinum treatment-generated resistance in SCLC patients. We propose that this animal model can be used for further studies on the mechanism of Cisplatin-resistance (Figure 2).

### TRIB2 was upregulated in cisplatin-resistant SCLC cells

**Figure 3:**
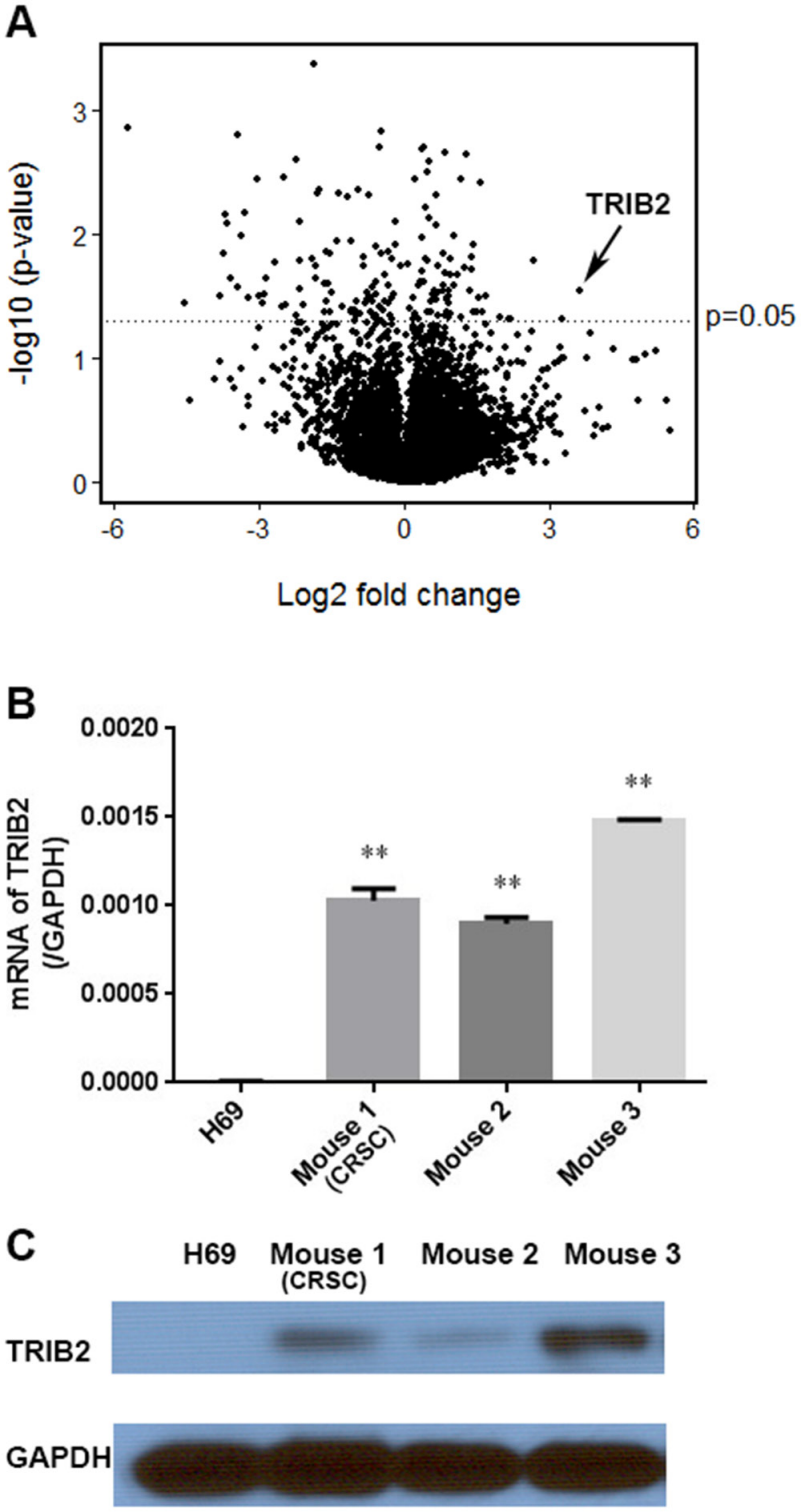
TRIB2 expression in Cisplatin-resistant SCLC cells **(A)** Volcano plot showing the relationship between the statistical p-values and the gene expression change ratio of CRSC vs. H69 cells in microarray analysis. **(B)** TRIB2 mRNA expression in H69 cells and SCLC cells from the three Cisplatin-treated mice. **(C)** TRIB2 protein in H69 cells and SCLC cells from the Cisplatin-treated mice. (TRIB2 gene indicated with arrow. (^**^, P < 0.01 vs. H69).

In order to evaluate the genetic alterations in the Cisplatin-resistant SCLC cells, gene expression profiles of CRSC and H69 cells were compared using microarray analysis. 162 genes with statistically significant expression changes, either increased or decreased, were identified (Supplementary Data 2). The expression levels in CRSC and H69 cells were compared and are shown in a Volcano plot (Figure 3A). Among those genes, 45 genes had expression changes of more than three-fold; among them, seven genes were upregulated and 38 genes downregulated (Supplementary Data 3). The genes with most significant expression changes were confirmed by RT-PCR (Supplementary Data 4). We selected TRIB2 for further study, both because it has been reported as an oncogene and because we found that the TRIB2 gene was upregulated the most among all the Cisplatin-treated mice (Supplementary Data 3) with p value < 0.05 (Figure 3A). RT-qPCR demonstrated that TRIB2 mRNA was expressed in the SCLC cells of all three Cisplatin-treated mice (0.0010, 0.0009 and 0.0015 compared to GAPDH expression), but was not detected in H69 cells (p < 0.01) (Figure 3B). Further, immunoblotting showed that TRIB2 protein was present in the SCLC cells of all three Cisplatin-treated mice, but was not evident in H69 cells (Figure 3C). This indicated that TRIB2 was significantly increased in the Cisplatin-resistant SCLC cells. (Figure 3).

### TRIB2 knockdown decreased cisplatin resistance

**Figure 4:**
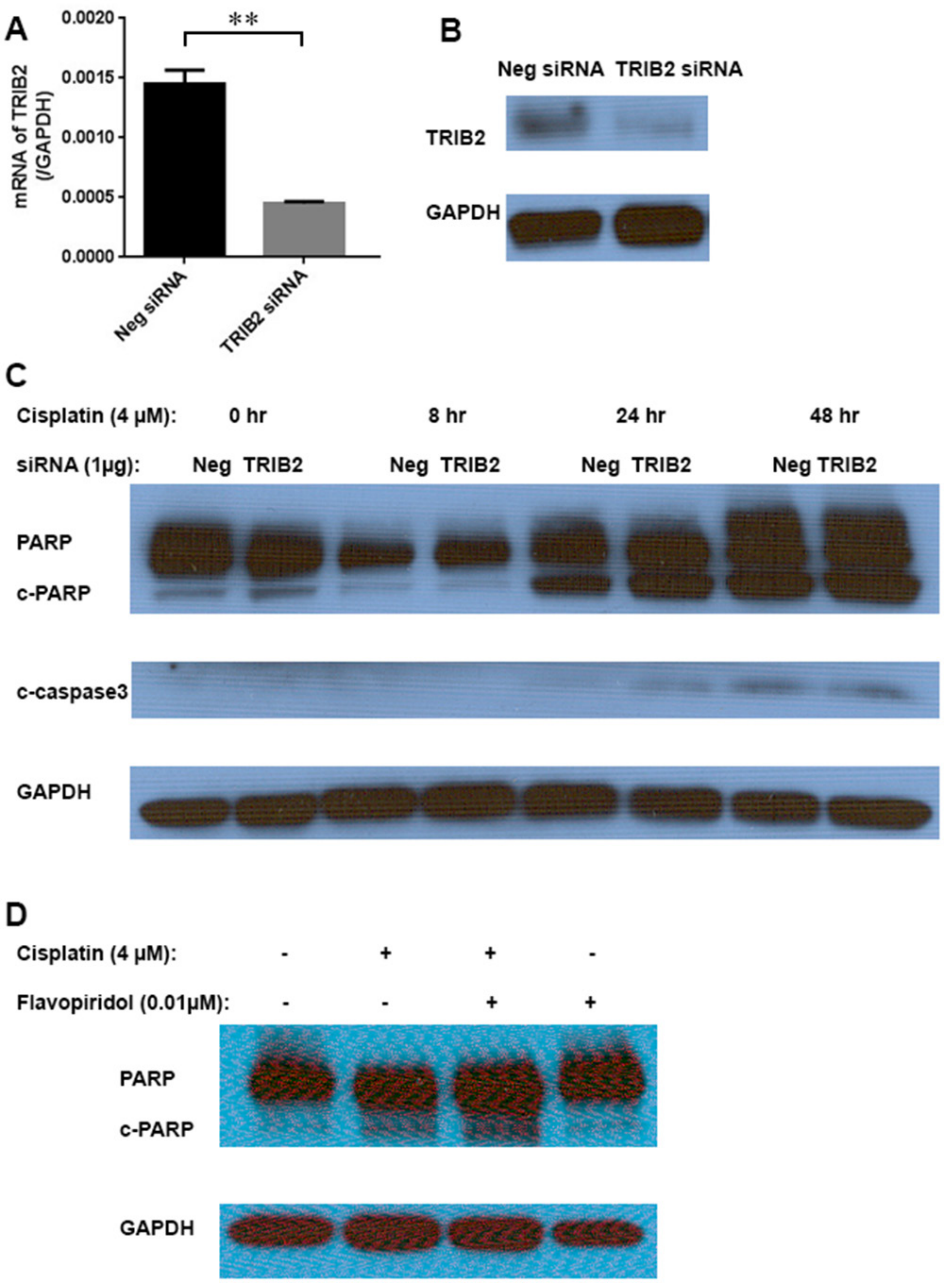
TRIB2 knockdown in Cisplatin-resistant SCLC cells **(A)** TRIB2 mRNA levels in CRSC cells transfected with either TRIB2 siRNA or negative siRNA (^**^, p < 0.01). **(B)** TRIB2 protein levels in the CRSC cells transfected with either TRIB2 siRNA or negative siRNA. **(C)** Immunoblot analysis of apoptotic markers during 48 hrs. Cisplatin-treatment course in the CRSC cells transfected with either TRIB2 siRNA or negative siRNA. **(D)** Immunoblot analysis of apoptotic marker in CRSC cells treated with Cisplatin and Flavopiridol for 24 hrs.

TRIB2 was the first member identified as an oncogene in the mammalian Tribbles (Trib) family of serine/threonine pseudokinases [24]. Overexpression of TRIB2 led to tumorigenesis of lung cancer [25], while downregulation of TRIB2 inhibited cell proliferation in cervical carcinoma [26] and lung cancer cells [27] [28]. In order to determine if the overexpressed TRIB2 contributed to Cisplatin-resistance, TRIB2 expression was knocked down in the CRSC cells using siRNA transfection. After confirming significantly reduced TRIB2 mRNA levels (∼70%) (Figure 4A) and obviously decreased TRIB2 protein level (Figure 4B), we found that higher levels of cleaved PARP and caspase-3 proteins were present in TRIB2 siRNA transfected CRSC cells than in negative siRNA transfected CRSC cells following treatment with 4 μM Cisplatin (Figure 4C). The most significant difference was seen at 24 hours after Cisplatin treatment. This result suggested that the overexpressed TRIB2 in the CRSC cells contributed to Cisplatin-resistance. We further tested whether TRIB2 protein inhibition could decrease the Cisplatin-resistance in CRSC cells by applying a kinase inhibitor, Flavopiridol, a protein kinase inhibitor which is known to decrease TRIB2 protein level *in vitro* [16]. After determining that 0.01 μM was a subtoxic (IC_10_) concentration of Flavopiridol in CRSC cells (Supplementary Data 5), we found that more cleaved PARP could be detected in CRSC cells treated with a combination of 0.01 μM Flavopiridol and 4 μM Cisplatin for 24 hours than either Flavopiridol or Cisplatin alone (Figure 4D). This result further supported the proposal that the overexpressed TRIB2 protein in the CRSC contributed to the Cisplatin-resistance and displayed the potential therapeutic benefits of the inhibition of overexpressed TRIB2 signaling in Cisplatin-resistant SCLC (Figure 4).

### TRIB2 downregulated CEBPA protein in cisplatin-resistant SCLC cells that acquired cancer stem cell-like properties

**Figure 5:**
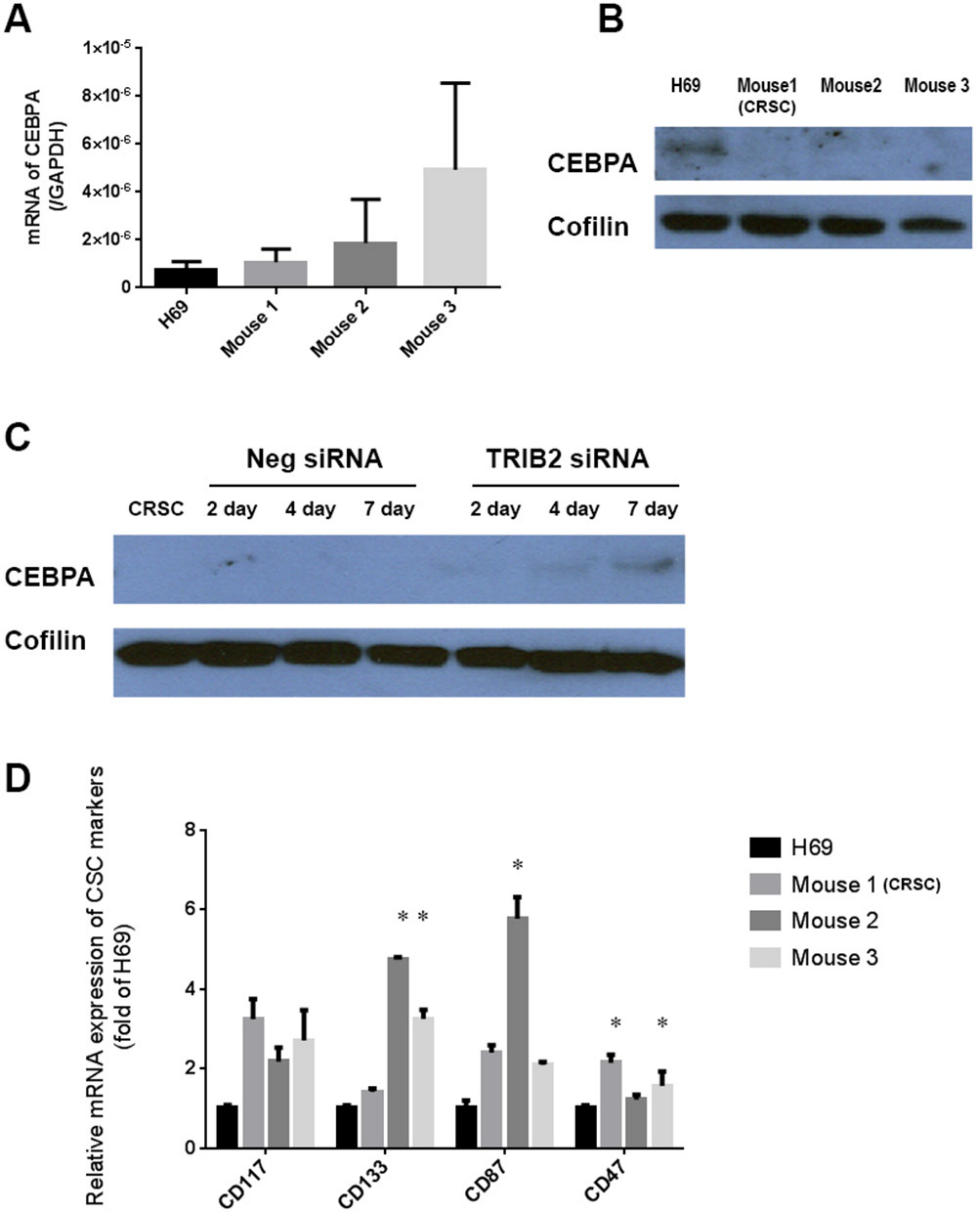
CEBPA and CSC markers in Cisplatin-resistant SCLC cells **(A)** mRNA of CEBPA in H69 cells and in SCLC cells from Cisplatin-treated mice (no statistical difference, p > 0.05). **(B)** CEBPA protein in H69 cells and in SCLC cells from Cisplatin-treated mice. **(C)** CEBPA protein levels in the CRSC cells transfected with TRIB2 siRNA and negative siRNA over seven days. **(D)** The mRNA expression of CSC markers in H69 cells and in the SCLC cells from Cisplatin-treated mice (^*^, p < 0.05 vs. H69).

Previous studies reported that TRIB2 could bind to the transcription factor CCAAT/enhancer binding protein alpha (CEBPA), leading to its degradation [29], [25], [30]. However, when detecting CEBPA expression, we noticed that the difference of the CEBPA mRNA expression levels in the SCLC cells from Cisplatin-treated mice and in H69 cells was not statistically significant (p > 0.05) (Figure 5A), whereas the CEBPA protein was undetectable in the SCLC cells from Cisplatin-treated mice, which was clearly detected in H69 cells (Figure 5B). Immunoblot analysis showed that CEBPA protein was re-induced in CRSC cells that were transfected with TRIB2 siRNA after 4-7 days (Figure 5C). This indicated that the downregulation of CEBPA protein in the Cisplatin-resistant SCLC cells resulted from the overexpression of TRIB2.

CEBPA was found initially to regulate normal hematopoietic developmental pathways and its abnormalities were identified in leukemia [31]. CEBPA silencing has been reported to block cell differentiation [32]. Considering that many chemo-resistant cancer cells have certain cancer stem-cell (CSC)-like properties and that these properties were inducible in the cancer cells [33], [34], [35], there was a possibility that the long-term Cisplatin treatment induced some CSC-like properties in the CEBPA-negative, Cisplatin-resistant SCLC cells. To determine whether this was true, we screened several CSC markers: CD117 and CD133 are identified as CSC markers of lung cancer [36], CD87 is associated with a CSC-like property in SCLC [37], and CD47 is highly expressed on virtually all human solid tumors, including lung cancer [38]. The mRNA expression levels of these CSC markers increased in the SCLC cells derived from the Cisplatin-treated mice; at least one of those markers significantly increased in the SCLC cell from each Cisplatin-treated mouse compared with H69 cells (CD47 in mouse 1; CD133 and CD87 in mouse 2; CD133 and CD47 in mouse 3) (p<0.05) (Figure 5D). Our results provide evidence that the Cisplatin-resistant SCLC cells acquired some CSC-like properties as a result of long-time Cisplatin treatment. Further study of the relationship between decreased CEBPA levels and the acquisition of stem-cell-like properties in the Cisplatin-resistant SCLC cells may be merited (Figure 5).

### TRIB2 and CEBPA protein was detected in pilot studies of human SCLC samples

**Figure 6:**
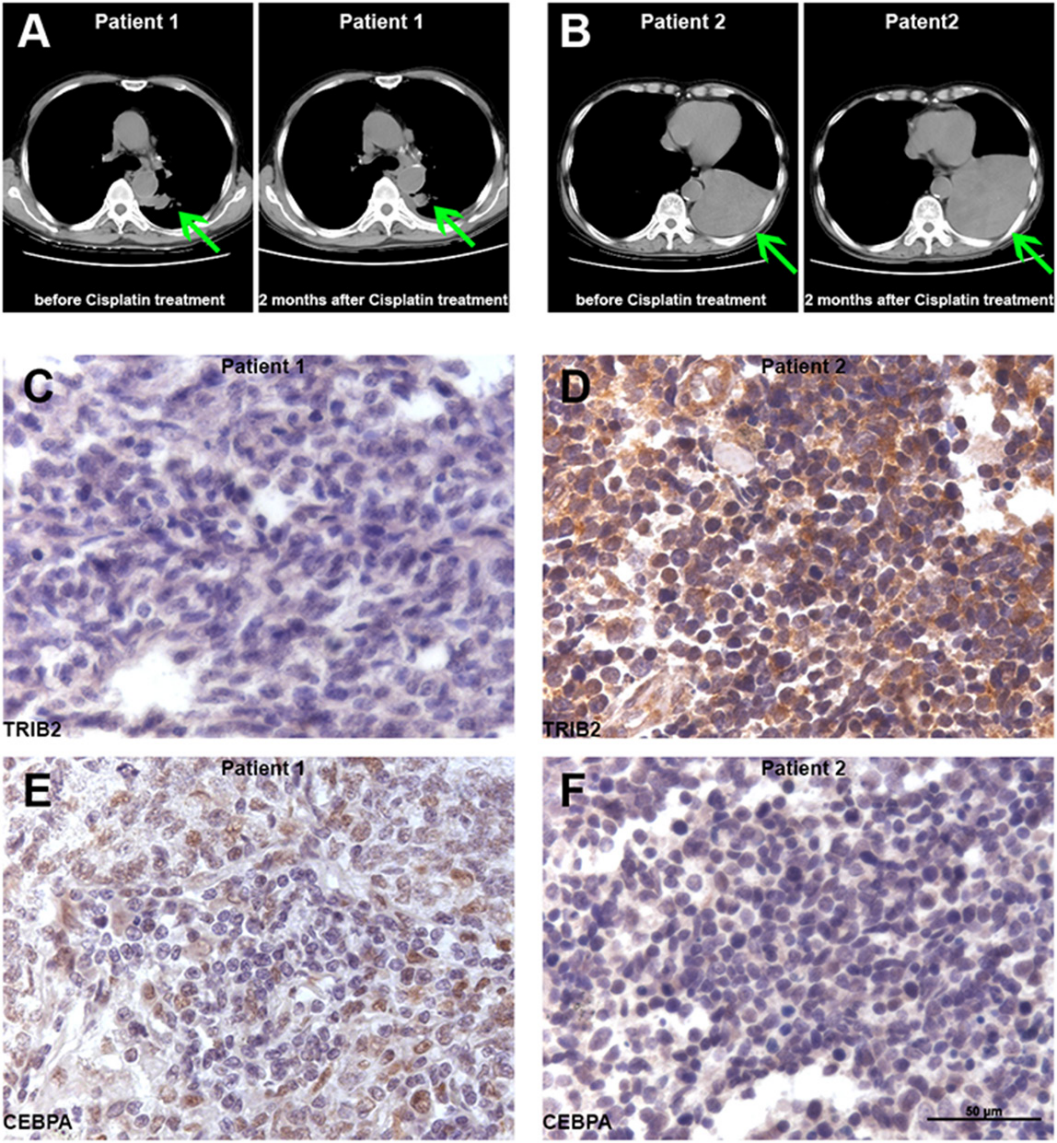
TRIB2 and CEBPA proteins in SCLC patients **(A)** Patient 1: CT scan before and two months after Cisplatin treatment. **(B)** Patient 2: CT scan before and two months after Cisplatin treatment. (Arrows indicate tumor.) **(C)** Tumor sample from the Cisplatin-sensitive Patient 1 did not express TRIB2 protein. **(D)** Tumor sample from the Cisplatin-resistant Patient 2 presented high levels of TRIB protein in the cytoplasm and nucleus of tumor cells. **(E)** Tumor sample from Patient 1 demonstrated CEBPA protein in the nucleus of the tumor cells. **(F)** Tumor sample from the Patient 2 did not have detectable CEBPA protein levels.

Clinical CT scanning showed that the size of the tumor in SCLC Patient 1 decreased after two months of chemotherapy that included Cisplatin (Figure 6A), but the size of the tumor in SCLC Patient 2 increased despite two months of Cisplatin-based chemotherapy (Figure 6B), indicating resistance of the latter. Thus, it appeared that the SCLC in Patient 1 was Cisplatin-sensitive, whereas the tumor in Patient 2 was Cisplatin-resistant. Immunohistochemistry (IHC) staining of the two tumor samples demonstrated CEBPA protein in the nucleus of tumor cells from the Cisplatin-sensitive patient, but not in those from the Cisplatin-resistant patient; further, TRIB2 protein was detectable in the cytoplasm and nucleus of tumor cells from the Cisplatin-resistant patient, but not in those from the Cisplatin-sensitive patient (Figure 6C-6F). The results of this limited patient pilot study are consistent with our observations in the Cisplatin-resistant H69 model: namely, that high levels of TRIB2 and low/nil levels of CEBPA were associated with Cisplatin-resistance. Although our SCLC patient samples are certainly limited, the TRIB2 and CEBPA proteins are very clearly identified in the specimens. Analysis of more tumor samples will be needed to definitively establish/verify a relationship between natural platinum resistance and TRIB2 and CEBPA protein expression. (Figure 6.)

## DISCUSSION

Since their introduction into the clinic more than three decades ago, platinum compounds have benefitted millions of solid tumor patients. Their use remains unabated in spite of recent advances that have made available a large number of new targeted and immunological therapies. Unfortunately, the great majority of cancer patients treated with platinum-containing regimens have some level of intrinsic resistance or develop acquired resistance to the drug and die of their disease within months. There is a vast literature dating back to the 1970’s reporting multiple mechanisms of natural and acquired resistance to platinum compounds. However, clinically effective interventions to reverse platinum resistance remain unavailable, probably because clinical resistance is multifactorial and many of the described resistance mechanisms may not be clinically relevant. In the last decade, the issue of platinum resistance has been addressed using empirical discovery approaches involving high-throughput screening technologies, but again practical advances in the treatment of this very important clinical problem remain elusive, probably for the same reasons [42].

Our study was designed to address this very old problem with a specific focus on small cell lung cancer (SCLC) using an *in vivo* model of platinum resistance. SCLC is a rapidly proliferating form of lung cancer characterized by a very high initial response rate to platinum containing chemotherapy followed by rapid development of resistance and a median survival of about one year. It is therefore one of the best disease models to study clinically relevant mechanisms of acquired resistance to platinum compounds. Unfortunately, tumor tissue non-availability is a major limiting factor in studying SCLC biology and developing rational molecular therapies, both at diagnosis before starting therapy and even more so after therapy, when resistance emerges. Therefore, studies with high-throughput screening technologies using clinical SCLC tumor specimens have rarely been performed. Most available literature of studies of platinum resistance in SCLC has been performed using cell lines in *in vitro* conditions. In view of the dismal impact of those studies on clinical practice, we decided to develop a model of SCLC with acquired *in vivo* resistance to Cisplatin and to use gene expression microarray analysis to identify new potential molecular mediators of resistance that might be used as therapeutic targets to reverse such resistance.

In this report, we have identified overexpression of the oncogene, TRIB2, as a potential mediator of acquired *in vivo* resistance to Cisplatin in SCLC, the first report of such overexpression being linked to Cisplatin resistance. This new observation is intriguing but biologically plausible in view of the existing literature on the oncogenic role of TRIB2 in acute leukemia and other malignancies [15], [16], [25], [26]. TRIB2 was the first member among the mammalian Tribbles (Trib) family of serine/threonine pseudo kinases identified as an oncogene. TRIB2 expression has been reported to mediate cell proliferation and cell survival and to be upregulated by the transcription factor E2F1. TRIB2 has been found to bind to the transcription factor CEBPA CCAAT/enhancer binding protein alpha (CEBPA), which represses the activity of E2F1, and to downregulate its expression by enhancing its proteasome-mediated degradation [25], [29], [30]. TRIB2 knockdown blocks malignant cell proliferation, thus confirming its role as an oncogene. Overexpression of TRIB2 leads to tumorigenesis of lung cancer [25], while downregulation of TRIB2 inhibits cell proliferation in cervical carcinoma [26] and lung cancer cells [27] [28].

We observed that NCI-H69 SCLC tumor xenografts chronically exposed *in vivo* to Cisplatin developed a 5-fold resistance to Cisplatin in association with overexpression of TRIB2 and downregulation of CEBPA, leading to enhanced proliferation and cell survival in the presence of the cytotoxic agent; further, that sensitivity could be restored at least partially by knocking down TRIB2, which results in CEBPA induction, or treatment with a cyclin-dependent kinase inhibitor, flavopiridol. The practical conclusion of our studies is that pharmacological inhibition of TRIB2 could sensitize those SCLC tumors which have a high expression of TRIB2 to Cisplatin. Finally, we analyzed the expression of both TRIB2 and CEBPA in two human samples of SCLC prior to therapy, one resistant and one sensitive to frontline platinum-containing chemotherapy; we observed high expression of TRIB2 and low expression of CEBPA in the resistant tumor and the opposite in the sensitive. These limited clinical observations remain anecdotal and we are expanding them to ascertain the role of TRIB2 in natural resistance to platinum compounds in SCLC.

The clinical relevance of our findings awaits corroboration in clinically relevant models. In view of the challenge in obtaining SCLC tumor tissue before therapy and upon progression to compare their expression of TRIB2, we have started to develop similar SCLC *in vivo* models of Cisplatin-resistance using patient-derived SCLC tumor samples. The clear advantage of these human tumor-derived xenografts is that they are more clinically relevant as they represent the multiclonality of the human disease and their vasculature and stroma remain partially of human origin for several passages [39]. Therefore, these models may unveil clinical resistance mechanisms not only induced by the presence of the cytotoxic agent, but also related to the emergence of resistant minority clones present at diagnosis as a result of selection pressure, which models of resistance derived from cell lines cannot identify.

## MATERIALS AND METHODS

### Generation of cisplatin-resistant SCLC cells *in vivo*

Numerous studies have developed drug-resistant human SCLC models, most of which have involved *in vitro* selection in the presence of the drug and have been performed in two-dimensional (flat) cultures [42], [43], [44]. However, complex cell/matrix interactions affect signaling pathways that can modulate drug sensitivity; thus, comparisons between *in vivo* responses of intact, three-dimensional tumors and such *in vitro*-selected drug-resistant models may not be well-aligned and informative. For these reasons, and despite the more protracted and resource-demanding approach, we undertook *in vivo* drug selection to develop Cisplatin-resistant SCLC models.

Six-to-eight weeks old athymic nude mice (21-27 g; male and female, Harlan) were implanted subcutaneously (s.c.) with NCI-H69 cells (ATCC); briefly, each mouse was injected into their right flank area with 5 x 10^6^ H69 cells suspended in 0.2 ml of serum-free RPMI medium. After obvious tumor lumps (> 2 x 2 mm) were observed, small biopsies were performed and the collected H69 cells from the mice were set as parental, non-drug treated H69 cells for further study. Then the mice were treated two to three times per week with tail vein injections of Cisplatin at a dose of 10 mg/kg per week. This has been the most effective therapeutic dose for SCLC and NSCLC mouse models in our preliminary studies. The injection protocol was maintained for 12 months unless: 1) The bodyweight of a mouse dropped to less than 18g, and/or 2) The tumor size became less than 2 x 2 mm. If bodyweight decreased to <18g or the tumor size decreased to <2 x 2 mm in a given mouse, injections were skipped until both the bodyweight rose to >18g and the tumor size became >2 x 2 mm.

Three mice out of 10 survived after the entire selection process: after a year of continuous i.v. injection of Cisplatin, all tumors in the surviving mice demonstrated Cisplatin resistance: thus, not responding to Cisplatin treatment. These tumors were harvested and the cells were cultured to select for the cancer cells over host’s normal cells. Briefly, the tumor was cut into small pieces (< 1.5 mm) and gently dispersed using a tissue grinder. The ground tissue was washed and suspended into the medium. Two days later the suspended and clustered cells were selected and transferred to fresh medium. This selection procedure was repeated every 2 days for 3 weeks. The adherent cells (majority of normal cells) and singly suspended blood cells were thus thoroughly excluded.

### Cell culture

Briefly described, H69 cells and the Cisplatin-resistant SCLC cells from the xenograft tumor (CRSC cells) were cultured in RMPI 1640 (Invitrogen) containing 10% fetal bovine serum (Invitrogen) in a humidified atmosphere of 5% of CO_2_ and 95% of air at 37°C. All cells used in this study went through the cell identification test. They 100% matched the profile of NIH-H69 from ATCC (Supplementary Data 1).

### Verification of cisplatin-resistant SCLC cells *in vivo*

To verify Cisplatin-resistance *in vivo*, first, the cultured SCLC tumor cells from 1-year Cisplatin-treated mice were implanted as before into nude mice and subjected to the same Cisplatin treatment protocol. Second, the resistance of the cultured SCLC tumor cells was compared with that of the parental cells H69 in the same mouse; Briefly, Female NOD-scid IL2Rgamma^null^ mice (Stock # 055557, Jackson Lab), 10-to-12 weeks old, were used for the second resistance verification test. An equal number (10^7^ cells/ mouse) of cultured SCLC tumor cells and parental H69 cells were implanted s.c. into SCID mice, separately (n=5). I.v. injection of Cisplatin (10mg/kg/week) started when palpable, significant tumor lumps were observed on both flanks. Tumor volume and body weight were measured twice a week. Tumor volumes were calculated using the greatest longitudinal diameter (length) and the greatest transverse diameter (width), as measured by external caliper. The modified ellipsoidal formula was used to express the tumor volume = 1/2(length × width^2^) [45] [46].

### RT-qPCR for gene expression

Total RNA was isolated from cultured cells by using RNeasy kit (Qiagen). cDNA was generated from about 2 μg total RNA by using SuperScript III Reverse Transcriptase (Invitrogen). qPCR was conducted with the Maxima Sybr qPCR mix (Fisher Scientific) in BioRad cfx96 real-time PCR detection system. The primers used are listed in Table 1. The mRNA level of GAPDH was used as an internal control.

**Table 1:** The primers

Primers	Forward	Reverse
TRIB2	CTTTTGCCTGTCTGCTCATAGT	ATAGCTTCGCTCAAAGAACACA
CEBPA	CAAGGCCAAGAAGTCGGTGGACAA	TCATTGTCACTGGTCAGCTCCAGC
ABCG2	ACGAACGGATTAACAGGGTCA	CTCCAGACACACCACGGAT
ALDH1A1	AACTCCTCTCACTGCTCTCCACG	GTCACCCTCTTCAGATTGCTTTTCC
THY1 (CD90)	TCAGGAAATGGCTTTTCCCA	TCCTCAATGAGATGCCATAAGCT
kit (CD117)	TACTCATGGTCGGATCACAAA	CCACTTCACAGGTAGTCGAGC
PROM1 (CD133)	AGTGGATCGAGTTCTCTATCAGTG	CAGTAGCTTTTCCTATGCCAAACC
PLAUR (CD78)	CCACTCAGAGAAGACCAACAGG	GGTAACGGCTTCGGGAATAGG
CD47	GGCAATGACGAAGGAGGTTA	ATCCGGTGGTATGGATGAGA
GAPDH	GGAGCGAGATCCCTCCAAAAT	GGCTGTTGTCATACTTCTCATGG

### Immuno-blot for protein level

The total protein and nuclear protein were extracted with Cell Lysis Buffer (Cell Signaling) and NE-PER Nuclear Protein Extraction Kit (Thermo Scientific). The collected proteins were separated by SDS-polyacrylamide gel electrophoresis and transferred to polyvinylidene fluoride (PVDF) membranes. The membranes were incubated overnight at 4 °C with primary antibodies of TRIB2 (1:1000, #13533), CEBPA (1:1000, #2295), PARP (1:1000, #9542), cleaved-PARP (1:1000, #5625), cleaved-Caspase 3 (1:1000, #9664), Cofilin (1:1000, #3312), and GAPDH (1:5000, #2118), all from Cell Signaling. After washing, the membranes were incubated for 2 hours at room temperature with anti-rabbit horseradish peroxidase-coupled secondary antibody (1:5000, GE Healthcare). The membranes were then developed with Amersham ECL Prime Western Blotting Reagent (GE Healthcare), and illuminated on premium autoradiography films (Denville Scientific).

### Alamar blue assay for cytotoxicity

The concentration of suspended cells was adjusted to 0.2 million cells/ml and added into 96-well plates (100ul/well). Six hours later, they were treated with Cisplatin or Flavopiridol at different concentrations and cultured at 37°C for 5 days. Then 10 μl Alamar blue solution (Bio-Rad) were added into each well. After 37°C incubation for 40 min, the fluorescence intensity, using excitation at 544 nm and emission at 590 nm, was measured with a FLUOstar Omega microplate reader (BMG Labtech), which correlated with cell survival. A cell viability versus drug concentration curve was generated and an IC_50_ was calculated by GraphPad Prism software.

### Microarray hybridization for gene expression

Total RNA was isolated from cultured cells by using an RNeasy kit (Qiagen), and submitted to the Genomics Core of Albert Einstein College of Medicine. The HuGene 1.0 microarray chip was selected as it is a whole transcript-based array for gene expression profiling including well-annotated exons based on RefSeq (www.ncbi.nlm.nih.gov/refseq), Ensembl (www.ensembl.org), and putative complete coding sequences from GenBank (www.ncbi.nlm.nih.gov/genbank) [47]. Briefly described: purified cRNA was fragmented, biotin labeled and then hybridized to an HG-133A microarray (Affymetrix) for 16 hours at 45°C, with rotating at 45 rpm in a GeneChip hybridization oven. Then the probe arrays were washed, stained, and scanned with a GeneArray® Scanner. GeneChip® Microarray Suite, version 4.01 (Affymetrix) was used to generate the subsequent data applied for statistical evaluation according to vendor’s instructions.

### siRNA transfection for mRNA knockdown

Parental H69 cells, or the cells from the tumor in mice treated with Cisplatin, were replenished with fresh medium and adjusted to a concentration of 0.5 million cells/ml one night before transfection. Amaxa Cell Line Nucleofector Kit T (Lonza) was used for transient transfection, following the electroporation method according to the vendor’s instructions. 1μg of TRIB2 siRNA (CGAAUUGCCUGGCUGAGUA) or a negative control (SIC001) (Sigma) was added to 1 million cells. The electroporation program A-023 was used; then the cells were cultured continuously and harvested for confirmation of TRIB2 knockdown by RT-qPCR two days later and by Western blotting four days later.

### Patient selection

Two SCLC patients from Hubei Provincial Corps Hospital, China, were enrolled in this study. The study was approved by institutional review boards of both Albert Einstein College of Medicine and Hubei Provincial Corps Hospital. Written informed consents were obtained from both patients. The selected patients met the following two criteria: 1) SCLC samples were collected from the patients; 2) clinical information was intact, including radiological evidence and at least 1-year follow-up. Detailed clinical information is presented in Table 2. Neither patient received any chemotherapy before the tumor sampling procedure; they received chemotherapy including Cisplatin only after tumor sampling.

**Table 2:** Patient information

Patient ^#^	Sampling procedure	Stage	Postoperative chemotherapy	Follow-up
1	Lobectomy + lymphadenectomy	T2N1M0	Etoposide+Cisplatin X6 cycles	1 yr follow-up did no found tumor by CT scan
2	Supraclavicular lymph node biopsy	T2N3M0	Etoposide+Cisplatin X2 cycles	The tumor was significantly progressed right after chemotherapy by CT scan

### Immunohistochemistry

Human SCLC specimens were fixed with 3.7% formaldehyde and embedded in paraffin. Cross sections were prepared and treated for 20 min at room temperature with 5% hydrogen peroxide to inhibit endogenous peroxidases. Slides were incubated overnight at 4°C with primary antibodies of TRIB2 (1:150, sc-100878, Santa Cruz) and CEBPA (1:100, #2295, Cell Signaling). Biotinylated anti-mouse and anti-rabbit secondary antibodies (Vector Laboratories) were added for 1 hour at room temperature to slides. The signal was amplified via the Elite Vectastain ABC kit (Vector Laboratories) and the peroxidase reaction performed at room temperature using DAB-substrate kit (Vector Laboratories) according to the vendor’s instructions.

### Statistical analysis

Values are presented as means and standard deviations (SD). The data were analyzed and graphed using Graphpad Prism 6. Unpaired, nonparametric t-test with two tails was used to compare the differences between the two groups; ANOVA (Analysis of variance) and multiple comparisons were used to compare differences among multiple groups. P < 0.05 was set as achieving statistical significance.

## CONCLUSION

A novel animal model was developed *in vivo* to generate Cisplatin-resistant SCLC cells from an established SCLC cell line, NCI-H69, and was used to confirm Cisplatin-resistance *in vitro* and *in vivo*. TRIB2 protein levels increased whereas CEBPA protein levels decreased in the Cisplatin-resistant SCLC cells, compared with their parental H69 cells. TRIB2 knockdown decreased Cisplatin-resistance and upregulated CEBPA protein. The Cisplatin-resistant SCLC cells also demonstrated certain cancer stem-cell-like properties, which may contribute to chemo-resistance in many cancers [40], [41]. This study revealed for the first time a possible molecular mechanism whereby TRIB2 overexpression contributed to Cisplatin-resistance by downregulating CEBPA protein levels in SCLC cells, and also suggested a potential therapeutic target for further study to overcome chemo-resistant SCLC.

## SUPPLEMENTARY MATERIALS






