# Supplementary material for: TRIB2 contributes to cisplatin resistance in small cell lung cancer
Source: Oncotarget. 2017 Nov 27;8(65):109596–608. doi: 10.18632/oncotarget.22741 (PMC5752545; doi:10.18632/oncotarget.22741)
Supplement: Supplementary file 1 [file oncotarget-08-109596-s001.pdf]

TRIB2 contributes to cisplatin resistance in small cell lung cancer

SUPPLEMENTARY MATERIALS

Supplementary Data 1: Cell line authentication allele report

See Supplementary File 1

Supplementary Data 2: Gene list and expression level

See Supplementary File 2

Supplementary Data 3: Gene list of expression changes more than 3-folds

Downregulated genes	Upregulated genes
MEP1B	ADCY6; MIR4701
SLC17A6	TADA3
ANO4	NETO1
SULT1E1	ZNF521
EPHA5	JUP
MPPED2	GTDC2
LRP1B	TRIB2
CCBE1	
PEG3; ZIM2	
RAB38	
KLHL41; BBS5	
CDH12	
GLT8D2	
EBF3	
SLN	
PRRG4	
ANKRD22	
PPEF1	
ASS1	
FEZ1	
SLC18A2	
ATXN1	
TCEA3	
GPR116	
EMP3	
PCDH11Y; PCDH11X	
GBP6	
DHRS3	
STAMBPL1	
PTPRD	
DNAJC22	
TUB	
PPP1R1B	
SYTL4	
NMNAT2	
MYBPC2	
SYNGR4	
ATP2B4	

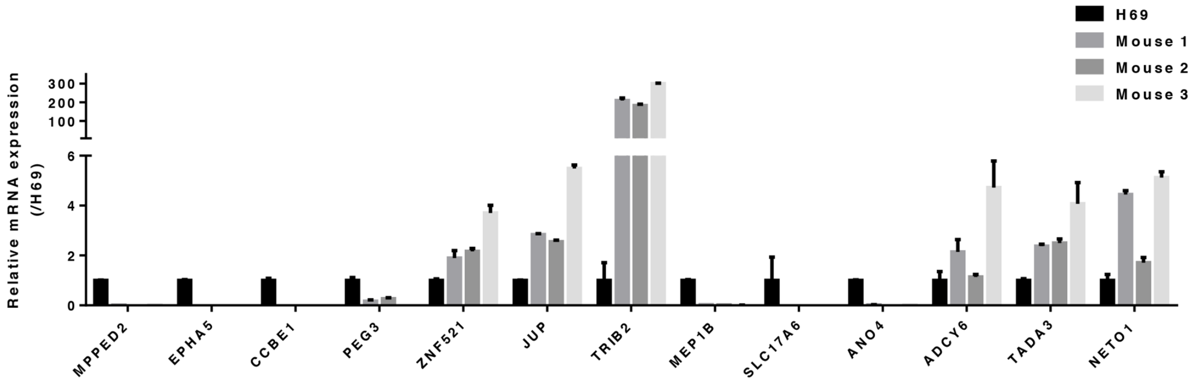

Supplementary Data 4: Relative mRNA expression folds of genes.

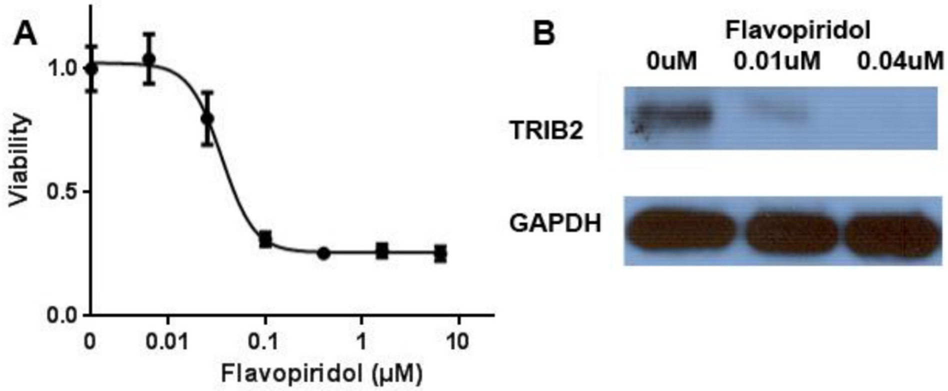

Supplementary Data 5: Cytotoxicity of Flavopiridol in the CRSC cells. Cytotoxicity of Flavopiridol was performed with Alamar blue assay. IC_{50} and IC_{10} of Flavopiridol were 0.03422 µM and 0.0138µM in the CRSC cells **(A)**. TRIB2 protein was decreased in the CRSC cells treated with Flavopiridol at the concentration of 0.01µM ($<IC_{10}$) **(B)**.

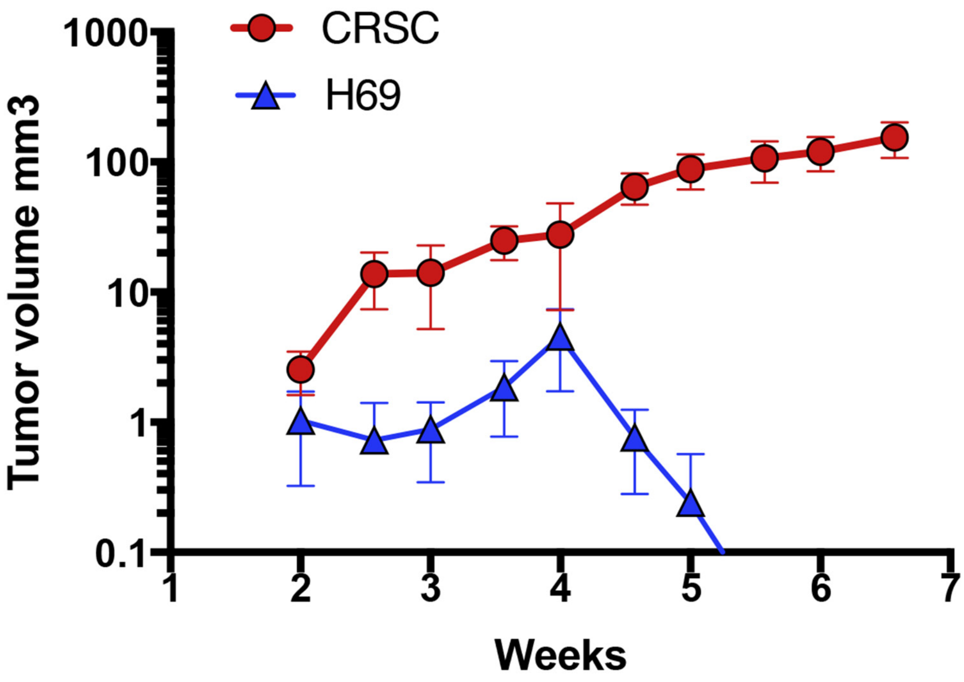

Supplementary Data 6: Cisplatin -resistance confirmation *in vivo* in same SCID mice. H69 and CRSC cells were simultaneously inoculated s.c. into the right and left flanks, respectively, of the same mice to ensure that the cells grew in similar environments and receive the same dose of treatment. Cisplatin injection started 4 weeks after the implantation.

Supplementary Data 7: Cell lines chosen for Cisplastin-resistance study

	[a]Cell CDDP tolerance	Tumorigenic	[b]Tumor CDDP tolerance	[c]Cell morphology; Tumor Histology	Original Location	Treatment before sampling
H69	N	Y	Y	Susp; T	Pleural effusion	Rad + chemi
H82	N	Y	N	Susp; V	Pleural effusion	Chemo
H209	N	Y	N	Susp; T	Bone marrow	No
H345	N	N	N/A	Susp; T	Bone marrow	Chemo
H378	N	Y	N	Susp; T	Pleural effusion	Chemo
H446	N	Y	N	Susp + adhe; V	Pleural effusion	Chemo
H526	N	Y	N	Susp; V	Bone marrow	No
H660	N	N	N/A	Susp; T	Lymph notes	No
SHP-77	N	Y	N	Susp & adhe; V	Lung	Radi & Chemo

The ideal *in vitro* model to study the Cisplatin-resistant mechanism is to select a cisplatin sensitive cell line and induce the chemoresistance using Cisplatin treatment; if succeed, the resistant cell line and parental cell lines will have the same genetic background.
We checked all of the following ATCC's SCLC cell lines. The creteira for selecting are: sensitive to high dose but tolerate to low dose of cisplatin, classical SCLC histology/morphology, and tumorigenecity. We got 9 cell lines from ATCC and tested all of them. Their characters and the test results of cisplatin tolerance and tumorigenecity are listed in the following table.

a: The tolerance means the cells can survive in the culture medium containing ≤ 0.1 μM of Cisplatin for ≥ 3 month.
b: The tolerance means the sc xenograft tumor (≥ 5 mm in diameter) in mice cannot be clear out by intravenous injections of cisplatin at 10 mg/kg/week in 2 month.
c: Where "T" and "V" means typical and variant SCLC, separately, based on the ATCC's description.
