# Supplementary material for: TRIB2 contributes to cisplatin resistance in small cell lung cancer
Source: Oncotarget. 2017 Nov 27;8(65):109596–608. doi: 10.18632/oncotarget.22741 (PMC5752545; doi:10.18632/oncotarget.22741)
Supplement: Supplementary file 2 [file oncotarget-08-109596-s002.docx]

| Report Style: Marker Table | |  |  |  |  |  |  |  |  |  |  |  |  |  |  |  |  |  |  |  |  |
| --- | --- | --- | --- | --- | --- | --- | --- | --- | --- | --- | --- | --- | --- | --- | --- | --- | --- | --- | --- | --- | --- |
|  |  |  |  |  |  |  |  |  |  |  |  |  |  |  |  |  |  |  |  |  |  |
|  |  | TH01 |  | D21S11 |  | D5S818 |  | D13S317 |  | D7S820 |  | D16S539 |  | CSF1PO |  | AMEL |  | vWA |  | TPOX |  |
| 1 | GM_GM0757_A01_CRSC_008.fsa | 8 | 9 | 30 | 31.2 | 11 | 13 | 12 | 12 | OL | OL | 11 | 11 | 10 | 12 | X | Y | 16 | 17 | 10 | 10 |
| 2 | GM_GM0757_B01_h69_007.fsa | 8 | 9 | 30 | 31.2 | 11 | 13 | 12 | 12 | 9 | 9 | 11 | 11 | 10 | 12 | X | Y | 16 | 17 | 10 | 10 |
| 3 | GM_GM0757_C01_ctrl+_006.fsa | 6 | 9.3 | 29 | 31.2 | 12 | 12 | 9 | 11 | 8 | 11 | 9 | 13 | 12 | 12 | X | Y | 16 | 19 | 11 | 11 |
| 4 | GM_GM0757_D01_ctrl-_005.fsa | 8 | 9 | 30 | 31.2 | 11 | 13 | 12 | 12 |  |  |  |  | OL | OL |  |  |  |  |  |  |
| 5 | GM_GM0757_E01_CRSC_004.fsa | 8 | 9 | 30 | 31.2 | 11 | 13 | 12 | 12 | 9 | 9 | 11 | 11 | 10 | 12 | X | Y | 16 | 17 | 10 | 10 |
| 6 | GM_GM0757_F01_h69_003.fsa | 8 | 9 | 30 | 31.2 | 11 | 13 | 12 | 12 | 9 | 9 | 11 | 11 | 10 | 12 | X | Y | 16 | 17 | 10 | 10 |
| 7 | GM_GM0757_G01_ctrl+_002.fsa | 6 | 9.3 | 29 | 31.2 | 12 | 12 | 9 | 11 | 8 | 11 | 9 | 13 | 12 | 12 | X | Y | 16 | 19 | 11 | 11 |
| 8 | GM_GM0757_H01_ctrl-_001.fsa |  |  |  |  |  |  |  |  |  |  |  |  |  |  |  |  |  |  |  |  |

**1. Cell line authentication allele report**

| % Match | Sample Count | Matches | Atcc Number | Designation | D5S818 | D13S317 | D7S820 | D16S539 | vWA | TH01 | AMEL | TPOX | CSF1PO |
| --- | --- | --- | --- | --- | --- | --- | --- | --- | --- | --- | --- | --- | --- |
| 100 | 14 | 14 | HTB-119 | NCI-H69 | 11,13 | 12 | 9 | 11 | 16,17 | 8,9 | X,Y | 10 | 10,12 |
| 100 | 13 | 13 | CRL-11351 | H69AR | 11,13 | 12 | 9 | 11 | 16,17 | 8,9 | X | 10 | 10,12 |
