# Supplementary material for: TRIB2 contributes to cisplatin resistance in small cell lung cancer
Source: Oncotarget. 2017 Nov 27;8(65):109596–608. doi: 10.18632/oncotarget.22741 (PMC5752545; doi:10.18632/oncotarget.22741)
Supplement: Supplementary file 3 [file oncotarget-08-109596-s003.docx]

**2. Gene list and expression level**

All listed genes show significant change (p<0.05) in expression and sorted by expression change fold.

| NAME | CRSC_1 | CRSC_2 | H69_1 | H69_2 |
| --- | --- | --- | --- | --- |
| MEP1B | 0.388251 | 0.34499 | 17.01727 | 22.3876 |
| EPHA5 | 0.629816 | 0.421799 | 5.998737 | 7.69877 |
| KLHL41; BBS5 | 0.520162 | 0.413119 | 3.96052 | 5.331819 |
| RAB38 | 0.827283 | 1.09781 | 8.035519 | 11.83205 |
| EBF3 | 0.25605 | 0.501606 | 2.592494 | 4.183261 |
| ANKRD22 | 0.153796 | 0.306642 | 1.479785 | 2.048585 |
| PPEF1 | 0.412557 | 0.734395 | 3.613366 | 4.452538 |
| SLC17A6 | 0.342715 | 0.729322 | 7.113356 | 18.69561 |
| SULT1E1 | 0.434169 | 0.393552 | 4.205853 | 7.915211 |
| CCBE1 | 0.567451 | 0.855374 | 6.036279 | 11.42901 |
| CDH12 | 3.898382 | 2.13558 | 19.24308 | 35.6203 |
| ANO4 | 0.716741 | 0.34499 | 6.16034 | 13.03779 |
| LRP1B | 0.424049 | 0.415992 | 3.247171 | 7.33414 |
| SLN | 0.471217 | 0.439711 | 2.494087 | 5.18602 |
| SLC18A2 | 2.447188 | 3.259449 | 11.33381 | 20.74408 |
| TCEA3 | 0.456654 | 0.729322 | 2.119786 | 3.46926 |
| MPPED2 | 0.525089 | 0.803644 | 8.300127 | 8.721812 |
| PEG3; ZIM2 | 0.723635 | 0.604841 | 7.329125 | 6.890592 |
| GLT8D2 | 0.797524 | 0.362142 | 5.871661 | 4.514693 |
| PRRG4 | 0.625759 | 0.630526 | 5.8177 | 4.514693 |
| ASS1 | 3.867276 | 6.040332 | 36.33682 | 28.93268 |
| PCDH11Y; PCDH11X | 0.419699 | 0.491284 | 2.298725 | 1.846288 |
| FEZ1 | 1.161789 | 1.323747 | 10.99218 | 5.331819 |
| EMP3 | 3.759217 | 3.259449 | 16.79229 | 15.83042 |
| ATXN1 | 0.353742 | 0.427687 | 2.068494 | 2.226269 |
| GBP6 | 0.27565 | 0.401822 | 1.414413 | 1.541813 |
| DNAJC22 | 1.65384 | 1.710749 | 5.887111 | 6.124652 |
| SYTL4 | 3.108531 | 3.64174 | 11.51458 | 11.11648 |
| MYBPC2 | 1.724696 | 2.487382 | 6.899574 | 6.340637 |
| GPR116 | 1.282384 | 0.82053 | 4.222875 | 5.635829 |
| PTPRD | 5.928335 | 4.805305 | 16.66401 | 22.07938 |
| GRM4 | 1.150446 | 1.596185 | 3.359327 | 4.577716 |
| CHODL | 1.809468 | 2.522104 | 5.556056 | 6.340637 |
| DOCK10 | 6.005516 | 4.906274 | 13.82875 | 14.26718 |
| VEGFA | 1.912811 | 1.808292 | 4.414166 | 4.9404 |
| IER5 | 6.01957 | 6.518897 | 12.31883 | 14.16863 |
| STAMBPL1 | 1.083067 | 0.855374 | 4.050385 | 3.19237 |
| DHRS3 | 2.860007 | 1.85913 | 11.62505 | 7.970266 |
| TUB | 3.917468 | 3.259449 | 14.9077 | 10.58998 |
| NMNAT2 | 5.296796 | 5.519846 | 21.87184 | 13.40434 |
| PPP1R1B | 1.765704 | 1.596185 | 6.10203 | 5.331819 |
| ITGA4 | 0.460915 | 0.388134 | 1.394873 | 1.144429 |
| CLCA4 | 0.318237 | 0.266947 | 0.91643 | 0.760293 |
| SYNGR4 | 1.016615 | 1.448568 | 4.334337 | 3.351084 |
| ATP2B4 | 2.127367 | 2.288858 | 7.798717 | 5.519846 |
| FBXO34 | 6.19007 | 6.082346 | 17.88462 | 12.50669 |
| DLG5 | 4.015889 | 4.483508 | 10.86418 | 7.915211 |
| NEK7 | 6.222726 | 5.481717 | 14.45339 | 10.8125 |
| TNNT1 | 4.383884 | 3.692577 | 9.187062 | 7.233169 |
| HOXA11 | 0.777825 | 0.843598 | 1.878766 | 1.73463 |
| SESN1 | 5.037448 | 6.253344 | 12.54909 | 10.08842 |
| HPN-AS1 | 0.367293 | 0.452073 | 0.860981 | 0.709379 |
| GK2 | 0.16417 | 0.212366 | 0.384261 | 0.359641 |
| GLRX | 1.247092 | 1.296505 | 2.504091 | 2.320809 |
| SMUG1 | 2.363981 | 2.837515 | 4.746237 | 4.241657 |
| TPP1 | 7.460938 | 6.795727 | 11.01925 | 13.12848 |
| ZNF700; ZNF763; OTTHUMG00000182304; CTD-2006C1.13 | 1.120551 | 1.269823 | 2.051552 | 2.210891 |
| KRTCAP3 | 1.337368 | 1.38956 | 2.231617 | 2.59301 |
| C11orf16 | 1.20785 | 1.2351 | 1.945488 | 2.077182 |
| PSPH | 8.484073 | 9.610609 | 13.63934 | 15.39754 |
| RFK | 2.238216 | 2.336952 | 4.195697 | 3.517689 |
| GLS2 | 2.583131 | 2.453137 | 4.459898 | 3.66707 |
| SLC45A3 | 0.940202 | 1.010191 | 1.629338 | 1.428626 |
| TMEM260; C14orf101 | 5.598844 | 5.519846 | 8.657102 | 7.915211 |
| ZMAT4 | 6.652118 | 7.084313 | 10.81471 | 9.677456 |
| ANXA7 | 16.33354 | 19.35491 | 27.66305 | 27.75408 |
| EIF2AK4 | 5.116439 | 6.210149 | 8.682622 | 8.661566 |
| SMPDL3A | 3.531012 | 4.012852 | 5.709317 | 5.406249 |
| MICU2 | 3.571149 | 4.183261 | 5.900737 | 5.519846 |
| WDR25 | 2.449128 | 2.522104 | 3.559629 | 3.517689 |
| SNAPC5 | 3.701943 | 3.66707 | 5.125059 | 5.009366 |
| E2F2 | 5.227367 | 5.29499 | 7.613302 | 7.035378 |
| C15orf32 | 0.30381 | 0.34739 | 0.452533 | 0.455217 |
| ZNF221 | 1.296184 | 1.448568 | 1.871645 | 1.783397 |
| TGIF2LY | 0.21825 | 0.24226 | 0.307557 | 0.300332 |
| RTN4IP1 | 1.803298 | 1.541813 | 2.428863 | 2.320809 |
| TMEM205; RAB3D | 7.096853 | 6.56424 | 10.30863 | 9.155432 |
| ST8SIA1 | 0.814674 | 0.781668 | 1.181135 | 1.024293 |
| SPRR2G | 0.215456 | 0.222925 | 0.274476 | 0.294151 |
| COG2 | 6.830106 | 6.429149 | 8.059868 | 8.601736 |
| GYPE | 0.307028 | 0.310923 | 0.394554 | 0.374913 |
| CYP4Z1 | 0.187821 | 0.195417 | 0.244864 | 0.230786 |
| PRSS37 | 0.378831 | 0.382791 | 0.46438 | 0.436674 |
| BNIP1 | 3.239657 | 3.236934 | 3.870989 | 3.718261 |
| SLC25A5 | 33.57946 | 34.40695 | 39.55914 | 42.95124 |
| ABCD4 | 2.485168 | 2.453137 | 2.79117 | 2.999304 |
| CYB5D1; OTTHUMG00000178176; RP11-1099M24.6 | 1.861818 | 1.95156 | 2.172473 | 2.180453 |
| OR5B2 | 0.164836 | 0.167778 | 0.186136 | 0.190073 |
| CXorf21 | 0.141145 | 0.142065 | 0.157733 | 0.160944 |
| S100G | 0.169425 | 0.168945 | 0.174486 | 0.17612 |
| PRAMEF10 | 1.080499 | 1.09781 | 1.01745 | 1.024293 |
| NDFIP2 | 6.753447 | 6.986781 | 6.162563 | 6.29684 |
| LENEP | 0.481815 | 0.494701 | 0.430893 | 0.410265 |
| NPY4R; LOC100996758 | 0.313094 | 0.294151 | 0.264969 | 0.265104 |
| CTTN | 7.817031 | 7.64559 | 6.431996 | 6.842995 |
| YWHAB | 11.82329 | 11.66915 | 9.786577 | 9.949527 |
| GALNT18 | 2.789269 | 2.539647 | 2.176706 | 2.120828 |
| ZFPL1 | 4.0087 | 3.90312 | 3.084703 | 3.374393 |
| ABCF3 | 17.39699 | 17.08464 | 13.28005 | 14.26718 |
| SGPL1 | 6.600475 | 6.38474 | 4.94689 | 5.29499 |
| LOC100507646 | 1.271936 | 1.305523 | 1.016161 | 0.96904 |
| ZFYVE1 | 2.828993 | 2.817915 | 2.143979 | 2.195619 |
| FAM117A | 0.952186 | 0.97578 | 0.724625 | 0.729322 |
| DHX16 | 9.031903 | 9.744768 | 7.207648 | 7.183206 |
| MDC1 | 13.39729 | 13.49757 | 10.48556 | 9.544224 |
| HK3 | 0.529225 | 0.545113 | 0.375171 | 0.404617 |
| MFSD1; LOC100287290; OTTHUMG00000158833; RP11-379F4.4 | 0.960177 | 1.010191 | 0.711533 | 0.709379 |
| GPATCH3 | 1.44369 | 1.563336 | 1.061893 | 1.09781 |
| NKIRAS1 | 2.399805 | 2.436192 | 1.737105 | 1.675542 |
| DPH2 | 7.574941 | 7.80624 | 5.347102 | 5.222092 |
| RBM14; RBM14-RBM4; RBM4; LOC101059993; OTTHUMG00000170428; RP11-658F2.8 | 17.71353 | 16.96663 | 11.503 | 13.2198 |
| TMUB2 | 5.189084 | 5.258415 | 3.294746 | 3.876159 |
| NBPF8; NBPF14 | 6.50894 | 6.167252 | 4.340956 | 4.483508 |
| TNK2 | 3.156504 | 2.958012 | 1.919808 | 2.180453 |
| TFE3 | 4.894091 | 4.483508 | 3.547927 | 3.170319 |
| H1F0 | 30.21563 | 26.62349 | 19.98284 | 18.43823 |
| NMU | 6.187662 | 6.082346 | 3.685024 | 4.012852 |
| METTL6 | 3.180991 | 3.170319 | 2.054903 | 1.924692 |
| DNAL4 | 4.713995 | 4.391239 | 2.946724 | 2.629207 |
| FAM86C1 | 4.828625 | 5.114623 | 3.405606 | 2.77912 |
| RASSF1 | 0.90335 | 0.765582 | 0.509039 | 0.537609 |
| SPHK2 | 2.089029 | 1.758844 | 1.177627 | 1.176603 |
| SALL2 | 4.265699 | 3.718261 | 2.273569 | 2.257347 |
| INPP5E | 4.722899 | 3.90312 | 2.428963 | 2.504683 |
| TUSC3 | 18.6566 | 15.72107 | 9.382145 | 9.610609 |
| KCNH6 | 2.217521 | 1.783397 | 1.066632 | 1.113134 |
| RSPH1 | 1.367878 | 1.120877 | 0.625217 | 0.734395 |
| SELK | 1.902666 | 1.911398 | 1.031523 | 1.090227 |
| NOVA1 | 0.471418 | 0.433657 | 0.221826 | 0.274452 |
| MAP4 | 5.178685 | 4.974763 | 2.466997 | 3.083626 |
| LAMB2 | 2.031501 | 2.034435 | 0.951815 | 1.128673 |
| SBK1 | 22.5865 | 20.74408 | 11.16257 | 9.8808 |
| SULT4A1 | 5.093756 | 4.125668 | 2.362749 | 2.13558 |
| LOXL3 | 3.868546 | 3.126672 | 1.548481 | 1.675542 |
| MTA1 | 9.505197 | 11.03969 | 6.518315 | 6.702168 |
| ASPHD2 | 3.356346 | 4.012852 | 2.141432 | 2.077182 |
| SETD2 | 13.76555 | 14.77031 | 9.001043 | 7.035378 |
| WDR82 | 29.95578 | 33.00535 | 18.65573 | 15.18556 |
| CCDC174 | 4.047615 | 4.641618 | 2.532682 | 2.048585 |
| USP4 | 16.86484 | 19.35491 | 8.386077 | 10.8125 |
| BSN | 3.216764 | 3.930269 | 1.784665 | 1.965134 |
| TJP2 | 8.997915 | 11.75032 | 5.039731 | 5.5969 |
| SUPT3H | 1.062624 | 1.287549 | 0.612784 | 0.580202 |
| SLC25A20 | 4.044907 | 5.079294 | 1.845189 | 1.992567 |
| PCDHB8 | 1.52253 | 1.675542 | 0.683264 | 0.704479 |
| TATDN2 | 20.16317 | 21.625 | 8.671689 | 8.251337 |
| BRPF1 | 7.431175 | 8.194341 | 2.49304 | 2.740859 |
| ETV4 | 2.840814 | 2.740859 | 1.135713 | 0.910435 |
| PLOD3 | 11.1977 | 7.80624 | 4.233748 | 4.183261 |
| RBM15B | 14.86631 | 12.59368 | 4.79123 | 5.714502 |
| ZNF787 | 8.32563 | 6.38474 | 2.660053 | 3.170319 |
| ZMIZ2 | 10.62846 | 7.915211 | 3.014929 | 4.012852 |
| RAF1 | 33.27202 | 26.62349 | 12.15128 | 10.3004 |
| ACP6; LOC101060704 | 1.787811 | 1.218096 | 0.48363 | 0.588302 |
| TADA3 | 12.46217 | 8.424715 | 3.029359 | 3.517689 |
| ADCY6; MIR4701 | 5.129156 | 3.445296 | 1.541446 | 1.176603 |
| NETO1 | 1.597577 | 1.184787 | 0.289772 | 0.481173 |
| ZNF521 | 13.79922 | 10.37204 | 3.580173 | 1.911398 |
| JUP | 17.87462 | 16.38868 | 2.202957 | 3.49339 |
| GTDC2 | 5.737003 | 3.66707 | 0.373842 | 0.873348 |
| TRIB2 | 5.167066 | 2.958012 | 0.271854 | 0.498142 |
| SALL3 | 3.179132 | 4.514693 | 1.164969 | 1.418757 |
